# Investigating the Structural Compaction of Biomolecules Upon Transition to the Gas-Phase Using ESI-TWIMS-MS

**DOI:** 10.1007/s13361-017-1689-9

**Published:** 2017-05-08

**Authors:** Paul W. A. Devine, Henry C. Fisher, Antonio N. Calabrese, Fiona Whelan, Daniel R. Higazi, Jennifer R. Potts, David C. Lowe, Sheena E. Radford, Alison E. Ashcroft

**Affiliations:** 1 Astbury Center for Structural Molecular Biology, School of Molecular and Cellular Biology, University of Leeds, Leeds, LS2 9JT UK; 2 Department of Biology, University of York, York, YO10 5DD UK; 3 Ipsen Ltd. UK, Wrexham Industrial Estate, 9 Ash Road North, Wrexham, LL13 9UF UK; 4 MedImmune, Sir Aaron Klug Building, Granta Science Park, Cambridge, CB21 6GH UK

**Keywords:** Ion mobility spectrometry, Electrospray ionization, Mass spectrometry, Proteins, RNAs

## Abstract

Collision cross-section (CCS) measurements obtained from ion mobility spectrometry-mass spectrometry (IMS-MS) analyses often provide useful information concerning a protein’s size and shape and can be complemented by modeling procedures. However, there have been some concerns about the extent to which certain proteins maintain a native-like conformation during the gas-phase analysis, especially proteins with dynamic or extended regions. Here we have measured the CCSs of a range of biomolecules including non-globular proteins and RNAs of different sequence, size, and stability. Using traveling wave IMS-MS, we show that for the proteins studied, the measured CCS deviates significantly from predicted CCS values based upon currently available structures. The results presented indicate that these proteins collapse to different extents varying on their elongated structures upon transition into the gas-phase. Comparing two RNAs of similar mass but different solution structures, we show that these biomolecules may also be susceptible to gas-phase compaction. Together, the results suggest that caution is needed when predicting structural models based on CCS data for RNAs as well as proteins with non-globular folds.

**Electronic supplementary material:**

The online version of this article (doi:10.1007/s13361-017-1689-9) contains supplementary material, which is available to authorized users.

**Graphical Abstract:**
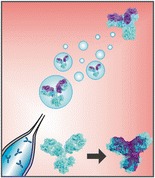
ᅟ

## Introduction

The advent of electrospray ionisation (ESI) transformed the field of mass spectrometry (MS) by providing the ability to routinely analyze not only large proteins but also noncovalently bound biomolecular complexes. In the three decades since this development, there has been a significant body of literature providing evidence of the native-like state of biomolecules measured by both ESI-MS and, more recently, ESI-ion mobility spectrometry-MS (ESI-IMS-MS) [1–3].

Ion mobility spectrometry (IMS) is a separation technique based on the gas-phase mobility of ions as they travel, under the influence of a weak electric current, through a drift tube filled with an inert gas [4–6]. Ions are separated based on their charge and shape: briefly, compact ions travel faster than extended ions carrying the same number of charges, whilst ions with a high number of charges travel faster than ions carrying a lower number of charges derived from the same precursor molecules. When coupled with MS, the data output is a 3D array of *m/z* versus intensity versus IMS drift time. The IMS drift time for ions can be converted to collision cross-section (CCS) directly if the IMS drift tube is a linear one [6–8], or indirectly following a calibration procedure [9–12] if the drift tube is of a traveling wave (TW) [13] design. The CCS of an ion corresponds to the averaged rotational 2D projection of the biomolecule’s 3D structure. Hence, ESI-IMS-MS is a unique and powerful tool that can separate and characterize biomolecules, providing both mass and shape (via CCS) information on individual species within an ensemble in a single, rapid, experiment. Indeed, ESI-IMS-MS has been employed to study the 3D architecture and conformational properties of many proteins and noncovalently bound biomolecular complexes [4–9, 14–22].

In 1997, Joseph Loo stated there are three camps of opinion concerning the retention of native protein structure upon transition into the gas-phase: “believers, nonbelievers, and undecided” [2], and quite possibly he was correct to hint at caution because despite the high number of successes reported, there has been a slow, low level emergence of literature demonstrating the “collapse” of certain proteins upon transition into the gas phase [23–25], one key example being antibodies [26–28]. Here, by systematic analysis of different non-globular proteins and RNA molecules using ESI-TWIMS-MS, we provide evidence of compaction in the gas-phase, highlighting a potential caveat in studying these specific biomolecules using this technique. The degree of compaction has been revealed by comparing the CCS values estimated from the ESI-IMS-MS data with CCS values calculated from the PDB structures of these biomolecules and also, in the case of the proteins, with in vacuo Molecular Dynamics (MD) simulations.

## Methods

### Biomolecules

The monoclonal antibody, mAb1, was supplied by Medimmune (Cambridge, UK); (I27)_5_ was expressed recombinantly as described elsewhere [29] with amino acid linkers in between each subunit (see Supporting Information, Supplementary Table S1); POTRAs were cloned and expressed recombinantly as described elsewhere [30]; a SasG construct (G5^1^-G5^7^-Strep-CysCys) was produced as described previously [31]. The RNA molecules were purchased from Integrated DNA Technologies BVBA, Leuven, Belgium.

### Protein Mass Spectrometry Analyses

All nanoESI-TWIMS-MS protein measurements were carried out using a Synapt HDMS mass spectrometer (Waters Corp., Wilmslow, UK). Samples were introduced to the mass spectrometer using in-house pulled borosilicate capillaries (Sutter Instrument Co., Novato, CA, USA) coated with palladium using a sputter coater (Polaron SC7620; Quorum Technologies Ltd., Kent, UK). All protein samples were analyzed in positive ESI mode. The *m/z* scale was calibrated using 10 mg/mL aqueous caesium iodide (CsI) clusters across the acquisition range (typically *m/z* 500–15,000).

Protein samples were dialyzed into 150 mM aqueous ammonium acetate before being infused into the Synapt HDMS instrument. nESI-MS and nESI-TWIMS-MS experiments were conducted under the following settings: capillary voltage, 1.5 kV; sample cone, 30 V; extraction cone, 4 V; source temperature, 60–80 °C; backing pressure, 3.0–5.0 mBar; trap voltage, 10–40 V; trap/transfer gas flow, 1.5 mL/min; IMS nitrogen gas flow, 20 mL/min, IMS wave height (ramped), 5–30 V; and traveling wave speed, 300 ms.

All data were processed and analyzed with the MassLynx v4.1 and Driftscope software, supplied with the mass spectrometer.

### ESI-TWIMS-MS CCS Calibrations for Proteins

ESI-TWIMS-MS experiments were carried out on a Synapt HDMS mass spectrometer using traveling wave IMS. Calibration of the traveling wave drift cell was carried out using a previously published method [11]. The calibrant proteins used were: beta-lactoglobulin, concanavalin A, alcohol dehydrogenase, and pyruvate kinase, taken from the Clemmer/Bush database [12]. Calibrant proteins were dissolved at a concentration of 10 μM in 200 mM ammonium acetate before being analyzed under the same conditions as the protein analytes.

Calibrant proteins were corrected for mass-dependant flight time using Equation 1 [11]:

1 $$ t{\prime}_D={t}_D-\left[\frac{C_{EDC}\sqrt{\frac{\mathrm{m}}{\mathrm{z}}}}{1000}\right] $$

where t’_D_ is the corrected drift time, t_D_ the measured drift time of the analyte, *m/z* the mass-to-charge ratio of the ion, and C_EDC_ the enhanced duty cycle (EDC) delay coefficient of the instrument (in this case 1.57). The corrected drift times were plotted against the reduced cross-sections (Ω’) as outlined in [11], and the plot fitted to a linear relationship (Equation 2):

2 $$ \varOmega^{\prime }= X\times \ln t{\prime}_D+ \ln A $$

where A is a fit determined constant and X the exponential factor.

The calibrations were converted to linear plots to allow for straightforward extrapolation for measurements of unknown proteins and complexes. For this, a new corrected drift time was calculated using Equation 3:

3 $$ t^{\prime }{\prime}_D= t{\prime_D}^X\times z\times {\left(\frac{1}{\mu}\right)}^{1/2} $$

where μ is the reduced mass of the ion.

The new corrected drift time (t′′_D_) was then plotted against the cross-section of the calibrant proteins (taken from the database [12]) to generate the calibration plots (see Supporting Information, Supplementary Figures S1, S2, and S3).

### RNA Mass Spectrometry Analyses

All nanoESI-TWIMS-MS RNA measurements were carried out using a Synapt G2-S mass spectrometer (Waters Corp., Wilmslow, UK). Samples were introduced to the mass spectrometer using in-house pulled borosilicate capillaries (Sutter Instrument Company, Novato, CA, USA) coated with palladium using a sputter coater (Polaron SC7620; Quorum Technologies Ltd., Kent, UK). All RNA samples were analyzed in negative ESI mode. The *m/z* scale was calibrated using 10 mg/mL aqueous caesium iodide (CsI) clusters across the acquisition range (typically *m/z* 500–15,000).

On receipt, the two 35-nucleotide RNAs (PDB structures 2PCV and 2DRB) were diluted to 200 μM in RNAse-free MilliQ water (Millipore UK, Watford, UK), separated into 100 μL aliquots and stored at –80 °C. When ready for analysis, these samples were diluted with 50 mM ammonium acetate to a concentration of 40 μM, desalted using spin columns (BioRad, Hemel Hempstead, UK) and then diluted to 8 μM with 50 mM ammonium acetate. nESI-TWIMS-MS experiments were conducted under the following settings: capillary voltage, –0.8 kV; sample cone, 30 V; source temperature, 60–80 °C; IMS sheath gas, He 2.42e-2 bar; IMS buffer gas, Ar 3.01 bar; IMS wave height 40 V; IMS wave velocity 400–750 m/s per scan.

### ESI-TWIMS-MS CCS Calibration for RNAs

ESI-TWIMS-MS experiments for the RNAs were carried out on a Synapt G2-S mass spectrometer using traveling wave IMS with negative ionisation electrospray. Calibration of the traveling wave drift cell was carried out using the method described previously in this document for protein samples but with an enhanced duty cycle delay coefficient (C_EDC_) of 1.41. The calibrant was a DNA polythymine of 10 nucleotides (d[T]_10_), the CCS of which had been measured and reported by Clemmer [32] (see Supporting Information, Supplementary Figure S4).

### Theoretical CCS Calculation

MOBCAL software was used to calculate the theoretical CCSs for the samples studied and was implemented using a Linux operating system. The MOBCAL projection approximation value [33] was used to generate the projection superposition approximation (PSA) as outlined in [34]. Equation 4 was used for this:

4 $$ P S A=\left( PA-81\right)\times 1.299 $$

### In Vacuo Molecular Dynamics (MD) Simulations

MD simulations were run using the NAMD software (NAMD 2.9), using the CHARMM force field [35]. Structures were simulated in a solvent-free system. For the simulation, a constant temperature of 300 K with Langevin thermostat was used and a time-step of 2.0 fs with a radial cut-off distance of 12 Å used throughout. Energy minimization in vacuo was implemented for a total of 0.5 ns before an equilibration of 10 ns; the cut-off distance, force field, and time step remained as described above throughout the simulation. Visual molecular dynamics (VMD) [36] was then used to visualize the simulation, and individual frames were saved as PDB coordinates in order to compute the CCS using MOBCAL. The VMD software was also used to calculate the root mean square deviation (RMSD) and radius of gyration (Rg). Analysis of the RMSD revealed whether a protein had equilibrated by the end of the 10 ns simulation; any sample that had not finished equilibrating was resubmitted for a further 10 ns until equilibration was reached. The NAMD and VMD software was operated under a Linux operating system.

## Results and Discussion

### Insights into the Gas-Phase Collapse of Monoclonal Antibodies

**Figure 1:**
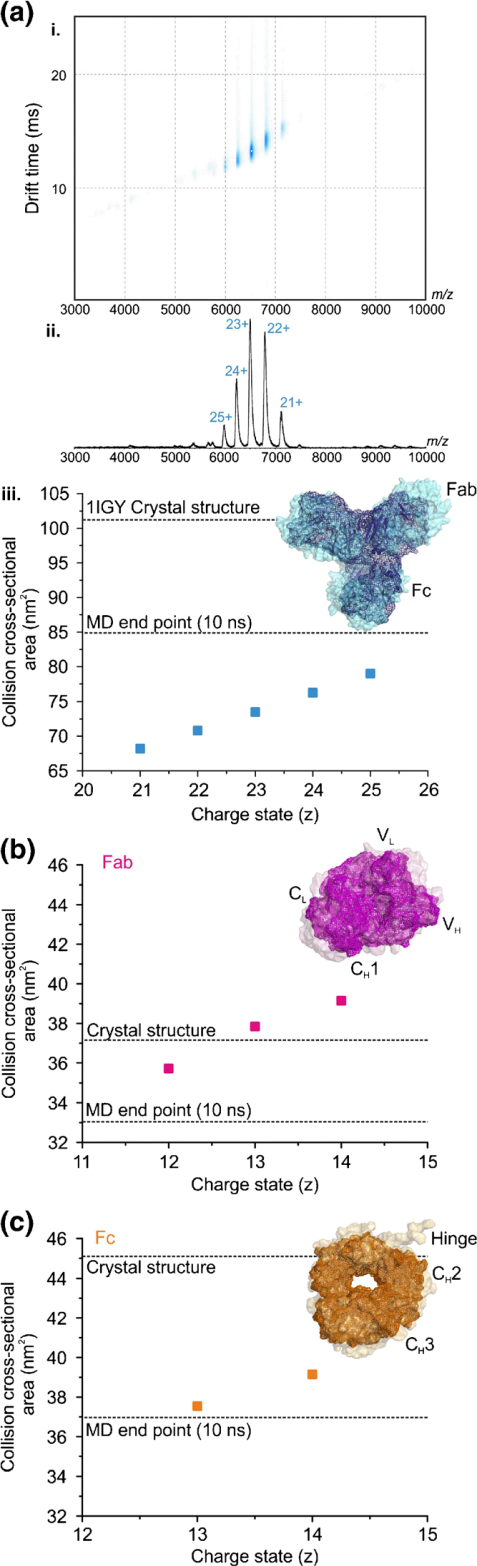
Monoclonal antibodies collapse around the flexible hinge region in the gas-phase. (**a**) ESI-TWIMS-MS (i) driftscope plot of *m/z* versus TWIMS drift time versus intensity, and (ii) mass spectrum for mAb1 with (iii) the TWIMS CCSs for each charge state compared with the CCS calculated from the protein’s PDB coordinates and the CCS estimated from the end-point of the in vacuo MD simulation. The ESI-TWIMS-MS CCS data for (**b**) the Fab and (**c**) the Fc regions of mAb1 isolated by Lys-C digestion: the calculated CCS values from the PDB coordinates and those after in vacuo MD equilibration are reported for both proteins (dotted lines). Insets show the PDB structures (transparent surface) alongside the in vac*uo* MD equilibrated structures (solid wire frame)

Using ESI-TWIMS-MS under non-denaturing conditions to characterise an IgG1 monoclonal antibody, mAb1, we observed that despite presenting a narrow ESI charge state distribution (21+ to 25+ ions) usually indicative of a “native-like” protein, the experimentally estimated CCS value of the lowest charge state (68.2 nm^2^) was significantly lower (32.4%) than the computationally determined CCS (101 nm^2^) based on the published structure (PDB = 1IGY [37]) (Figure 1a i, ii, iii). Similar behavior of monoclonal antibodies (mAbs) has been reported by others [27, 28], and Pacholarz et al. carried out in vacuo MD simulations to interrogate the observed compaction of IgG molecules in the gas-phase, demonstrating that the protein likely collapsed around the hinge region in between the fragment antigen-binding (Fab) and the fragment crystallizable (Fc) regions [28].

Molecular modeling is a useful tool to aid the study of biomolecules in the gas-phase. Although CCSs measured using ESI-TWIMS-MS methods can be compared directly to solved X-ray crystal or NMR structures from the Protein Data Bank (PDB), it is becoming clearer that this is not suitable for all proteins. For example, the conditions used to crystallize proteins can be very different to the conditions used for mass spectrometric analysis. Further, some proteins are inherently flexible or disordered, and may not have a PDB structure with which to compare the measured CCS, and additionally a subset of structures in the PDB consist only of fragments of the full protein in question. In vacuo modeling, therefore, allows us to achieve a glimpse of how such proteins may behave within the gas-phase. Adopting a similar in vacuo MD simulation approach as used by [28], we also observe a collapse around the hinge region of mAb1 such that the measured CCS of the mAb is substantially less than both the predicted CCS from its crystal structure [37] and the in vacuo MD simulation (Figure 1a iii).

To understand the role of the hinge region and determine whether this flexible linker was the main attributor to the collapse observed, we released the Fab and Fc regions of mAb1 using Lys-C proteolysis and analyzed the two fragments independently (Figure 1b, c). The CCS values determined by ESI-TWIMS-MS, estimated from the PDB coordinates, and indicated by in vacuo MD simulations are compared for both the Fab and the Fc regions (Figure 1b and c, respectively). The MD simulations indicated that both proteins collapsed to some extent in vacuo compared with their crystal structures, with the Fab region collapsing 11% compared with the 17% collapse of the Fc region. Furthermore, the CCS of the Fab region measured by ESI-TWIMS-MS was closer in agreement to the CCS predicted from its PDB structure than with its equilibrated collapsed MD structure, whereas the CCS of the Fc region measured by ESI-TWIMS-MS was closer to that of its collapsed MD structure than with its crystal structure. Although both of these fragments consist of four Ig domains, the Fc region retains the majority of the hinge region, supporting the notion that the flexible hinge plays a prominent role in the gas-phase collapse observed.

### Investigating the Gas-Phase Collapse of Other Nonglobular Proteins

**Figure 2:**
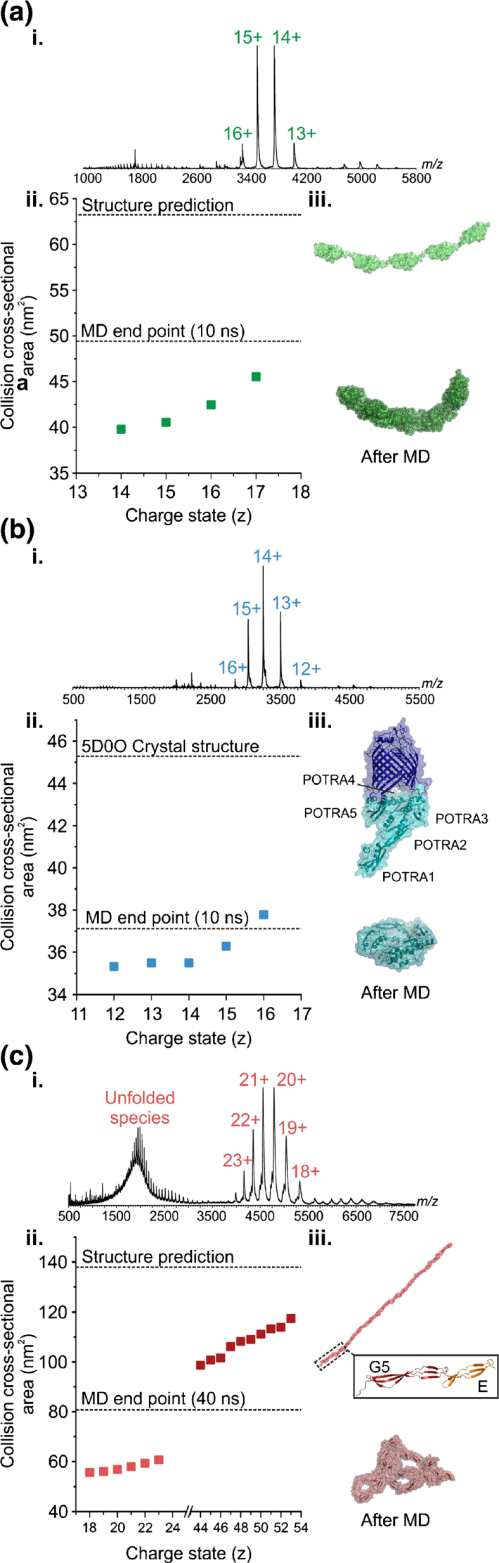
Compaction in the gas phase is observed for a range of non-globular proteins: (**a**) (I27)_5_, (**b**) BamA POTRA domains, and (**c**) SasG. ESI-TWIMS-MS (i) mass spectra, and (ii) CCS data for each protein are shown together with the theoretical CCS values before (from the PDB coordinates) and after in vacuo MD equilibration (dotted lines). The starting and equilibrated structures are shown for comparison

To investigate the generality of the role of flexible hinge regions in gas-phase protein collapse, using ESI-TWIMS-MS we analyzed an I27 concatamer, (I27)_5_ [29] (Figure 2a) and the POTRA domains from BamA [38] (Figure 2b). (I27)_5_ is a mechanically robust pentamer, the folded Ig subunits of which are connected by flexible linkers of 4–6 amino acids. This construct is used widely for AFM and mechanical stability studies [29, 39]. Furthermore, poly-Ig domains as well as I27 polyproteins have been shown to be flexible in solution and can adopt various conformations as revealed by electron microscopy [39–41]. The POTRA domains were chosen as, similar to (I27)_5_, the protein consists of five subunits (POTRAS 1–5) connected by short linker regions [42, 43].

The mass spectrum of (I27)_5_ indicated a narrow charge state distribution (13+ to 16+ ions) (Figure 2a i). As neither a crystal nor NMR structure was available for (I27)_5_, a model was built based on the solution structure of the I27 monomer (1TIT, [44]) and building in the 4–6 residue linker regions (see Supporting Information) (Figure 2a i). This enabled a theoretical CCS for the five-domain construct to be established and formed the starting point for the in vacuo MD simulations (see Supporting Information). The measured CCS for (I27)_5_ (39.8 nm^2^) [45] is lower than both the modeled value predicted for the native structure (63.1 nm^2^) and the MD simulation end point (49.4 nm^2^) (Figure 2a ii). Upon in vacuo minimization and equilibration, the protein undergoes compaction, then collapses around the flexible linker regions between the individual subunits, which is reflected by the CCS at the end of the simulation.

ESI-TWIMS-MS analysis of the combined POTRA domains from BamA again produced a mass spectrum with a narrow charge state distribution (12+ to 16+ ions) (Figure 2b i). The ESI-TWIMS-MS data indicate that the CCS (35.1 nm^2^) obtained for the lowest charge state ions (12+) is closer to the predicted CCS of the in vacuo-equilibrated structure (37.1 nm^2^) than the CCS value predicted from the crystal structure (5D0O; 45.3nm^2^) (Figure 2b ii). The MD collapse observed for the POTRA domains is attributable to compaction around the short hinge regions between the individual domains, as well as to an overall collapse with POTRA1 moving towards POTRA5, resulting in a more ring-like structure in the equilibrated molecule (Figure 2b iii).

Together, the data presented for mAb1, (I27)_5_, and the POTRA domains suggest that non-globular proteins with flexible linker or hinge regions are susceptible to gas-phase collapse. To determine how linear, elongated molecules without any distinct linker regions behave upon transition to the gas phase, we studied the protein SasG (Figure 2c). SasG consists of repeats of two domains (G5 and E), in which the C-terminus of any given domain is directly connected to the N-terminus of the subsequent domain (Figure 2c). Furthermore, SasG (G5^1^–G5^7^) has been shown to form long, elongated fibrillary structures that maintain a highly extended conformation in solution, with no evidence of compaction [31]. The ESI-MS data indicate a native-like conformation, centered on the 20+ and 21+ charge state ions, together with a highly charged, more unfolded conformation (centered on the 48+ charge state ions) (Figure 2c i). The ESI-TWIMS-MS CCS of the compact conformation was measured at 57.7 nm^2^ (18+ ions). In comparison, the predicted CCS based on the structure obtained from SAXS data [43] was 137.8 nm^2^, whereas the in vacuo MD simulations indicate that the protein collapses in the absence of solvent to a species with a CCS of 80.7 nm^2^ (Figure 2c ii, iii). Thus, an elongated linear protein, with no obvious linker or hinge regions, can also undergo significant compaction in the gas-phase.

### Gas-Phase Collapse of Other Biomolecules

**Figure 3:**
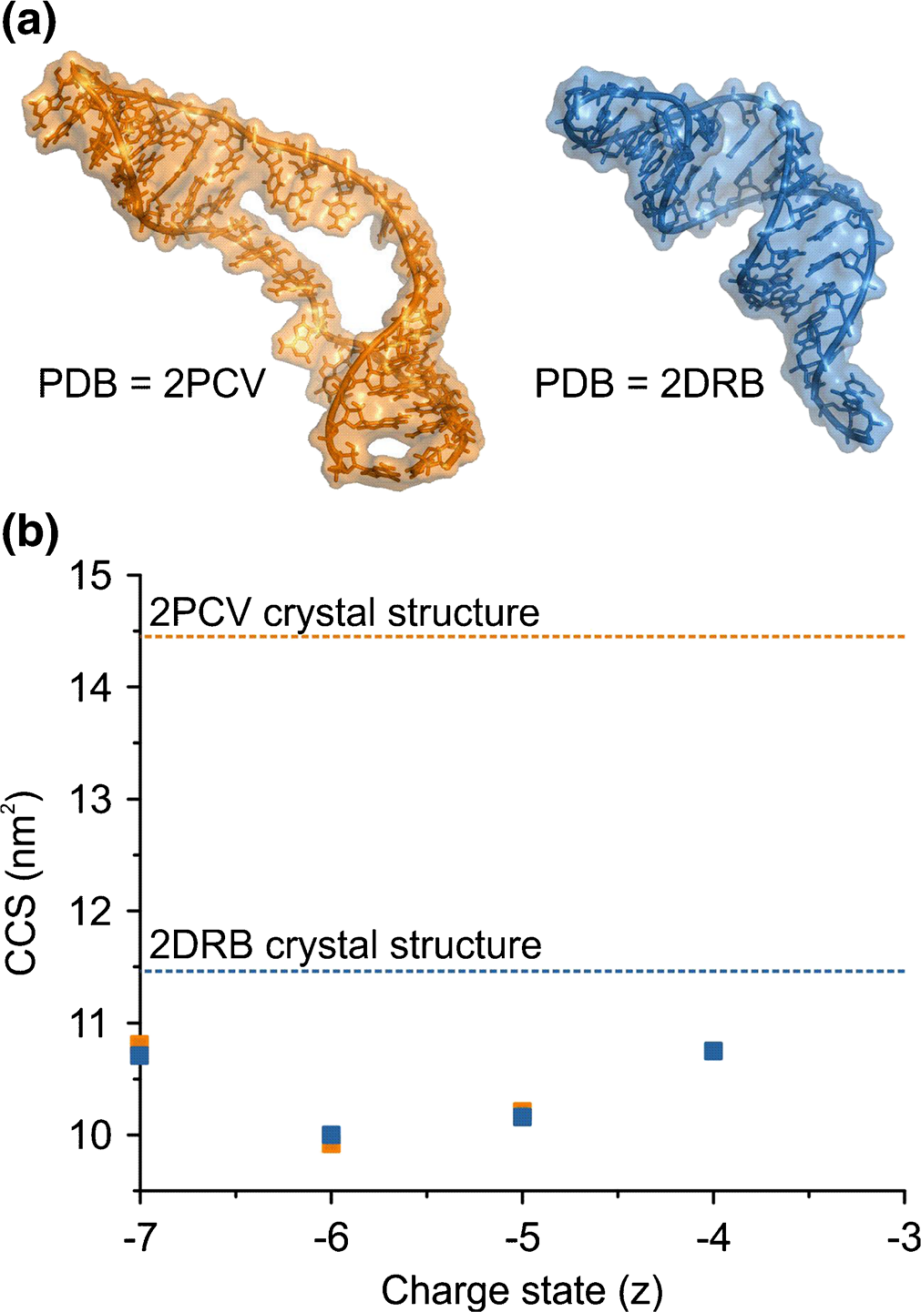
Observed gas-phase collapse of RNAs. (**a**) The structures of two 35-nucleotide RNA molecules: 2PCV [48] (orange) and 2DRB [49] (blue). (**b**) ESI-TWIMS-MS CCS data for the 4- to 7-charge state ions of the two RNAs, together with the predicted values derived from the respective PDB coordinates of the RNAs (dotted lines)

Recent ESI-TWIMS-MS studies indicated that the DNA duplex [d(GCGAAGC)] is a dynamic ensemble in the gas phase [46], in contrast to earlier work on G-complexes of ≥20 nucleotides, which suggested that their chemical topology remained unaltered in the gas-phase [47]. Here, we carried out ESI-TWIMS-MS analyses on two RNAs, each of 35 nucleotides and of very similar mass, but different sequences and secondary structures (2PCV [48]; 11,217 Da and 2DRB ([49]; 11,219 Da) (Table 1; Figure 3a) to determine if their 3D structures were preserved in the gas phase and hence if it was possible to differentiate between the two. An NMR solution structure has been published for 2PCV [48] and a crystal structure for 2DRB [49], and these were used to calculate CCS values (Figure 3b).

**Table 1:** The Molecular Masses, Sequences, and Predicated CCSs of the Two 35-Nucleotide RNAs: 2PCV and 2DRB

RNA	Mass	Mobcal PA	Sequence
2PCV	11,217 Da	1445 Å^2^	5′- GGA CCC GCC ACU GCA GAG AUG CAA UCC AGU GGU CC -3′
2DRB	11,219 Da	1146 Å^2^	5′- GGC CCG GGG CGG UUC GAU UCC GUU CUG GGC CAC CA -3′

ESI-TWIMS-MS analysis of the RNAs yielded identical CCSs for all of the corresponding charge state ions (4- to 7-ions; CCS ~10–11 nm^2^) (Figure 3b). Comparing the TWIMS CCS values with the CCSs estimated from the PDB structures, the TWIMS CCS data were significantly lower than the predicted values for either 2PCV (14.45 nm^2^) or 2DRB (11.46 nm^2^). For example, in the case of the 5-ions, TWIMS CCSs of 10.21 nm^2^ for 2PCV and 10.16 nm^2^ for 2DRB were measured, thus indicating both RNAs undergo gas-phase collapse. It may be argued that the ESI-MS solution conditions (50 mM aqueous ammonium acetate) differ from the crystallography conditions used for 2DRB (50 mM HEPES, 80 mM ammonium sulfate [n.b. some crystals were detected in the absence of the sulfate ions], 0.2 M tri-lithium citrate, and 20% PEG4000 [49]) and from the NMR solution conditions used for 2PCV (5 mM cacodylate, 50 mM NaCl, and 0.1 mM EDTA [48]), and that this may have affected the CCS values obtained from the three biophysical techniques. Although beyond the scope of this study, a systematic analysis of the effects of counter-ions, pH, oligonucleotide length, and sequence on collapse in the gas-phase with parallel MD simulations [46, 50] could be informative to cast more light on the response of RNA molecules in the gas-phase in general. However, here the collapse of both of the RNAs to a similar degree in the gas-phase is evident.

## Conclusion

The question remains: can the solution structure of proteins be retained upon transfer into the gas phase? For stable, globular proteins, the answer is undoubtedly “yes,” backed by an impressive number of literature examples. However, here we have presented a small number of protein examples from our 14 years’ experience with ESI-IMS-MS where we have found that the CCS values measured underestimate the physical size of the solution structure and modeled data of the biomolecule under scrutiny. This phenomenon has been reported elsewhere in the case of antibodies [26–28], but here we have shown by studying isolated regions of an antibody that the Fc region, which contains the majority of the flexible hinge region, is more prone to gas-phase compaction than the Fab region. Other proteins we have identified that undergo gas-phase compaction include those with flexible hinge regions in between more structured domains, such as an engineered concatamer, (I27)_5_, in addition to the BamA complex with its extended array of POTRA domains. Other non-globular proteins such as SasG, an elongated linear protein, can also exhibit this behavior. Gas-phase compaction is not limited to proteins, as illustrated with reference to two 35-nucleotide RNA molecules of similar mass but different shape. Both RNAs appeared from the ESI-TWIMS-MS data to be significantly smaller than expected from their 3D crystal or solution structures.

We do not intend this report to be perceived as a negative message to the use of ESI-TWIMS-MS. Indeed, the advantages of this technique far outweigh any disadvantages. However, there are certain classes of biomolecules for which due caution should be employed when interpreting the results.

## Electronic supplementary material

Below is the link to the electronic supplementary material.

ESM 1 (DOCX 196 kb)
