# Supplementary material for: Investigating the Structural Compaction of Biomolecules Upon Transition to the Gas-Phase Using ESI-TWIMS-MS
Source: J Am Soc Mass Spectrom. 2017 May 8;28(9):1855–62. doi: 10.1007/s13361-017-1689-9 (PMC5556138; doi:10.1007/s13361-017-1689-9)
Supplement: Supplementary file 1 — (DOCX 196 kb) [file 13361_2017_1689_MOESM1_ESM.docx]

**Investigating the structural compaction of biomolecules upon transition to the gas-phase using ESI-TWIMS-MS**

**Paul W. A. Devine^1^, Henry C. Fisher^1^, Antonio N. Calabrese^1^, Fiona Whelan^2^, Daniel R. Higazi^3^, Jennifer R. Potts^2^, David C. Lowe^4^, Sheena E. Radford*^1^, Alison E. Ashcroft*^1^**

^1^Astbury Centre for Structural Biology, School of Molecular and Cellular Biology, University of Leeds, Leeds, LS2 9JT, UK

^2^Department of Biology, University of York, York, YO10 5DD, UK

^3^Ipsen Ltd. UK, Wrexham Industrial Estate, 9 Ash Road North, Wrexham, LL13 9UF, UK

^4^MedImmune, Sir Aaron Klug Building, Granta Science Park, CB21 6GH, Cambridge, UK

**Supporting Information**

**Generation of the (I27)_5_ concatamer structure**

The I27 monomer subunit structure was taken from the PDB (PDB 1TIT). The four linker domains connecting the subunits were then attached to the C-terminus of the previous sub-unit based upon the linkers used in the recombinant proteins; the linker regions added are shown in Table S1.

| Linker | Amino acid composition |
| --- | --- |
| Linker 1 | VEAR |
| Linker 2 | LIEAR |
| Linker 3 | LSSAR |
| Linker 4 | LIEARA |

*Table S1: Table of amino acid linkers added to the C-terminal of the I27 sub-units 1-4.*

The five PDB structures were then aligned manually with one another before the C-terminal amino acid from the linker region was connected to the N-terminal leucine of the sequential I27 sub-unit. The subunits were connected using the Coot software (1), operated under a Linux operating system.

**ESI-TWIMS-MS Collision Cross-Section (CCS) calibrations for proteins (2,3)**


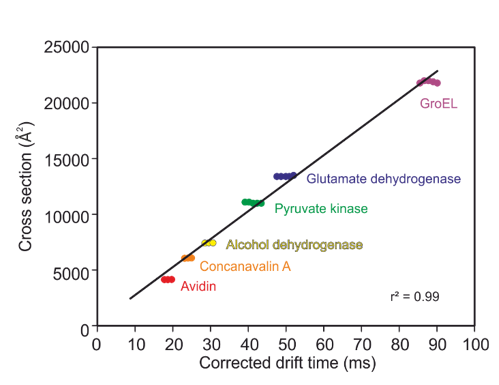


Figure S1: For mAb sample analysis, the TWIMS cell was calibrated using previously reported calibrant proteins (3). The plot shows avidin (15+ to 17+ charge states), concanavalin A (19+ to 21+ charge states), alcohol dehydrogenase (23+ to 25+ charge states), pyruvate kinase (31+ to 35+ charge states), glutamate dehydrogenase (37+ to 41+ charge states) and GroEL (65+ to 69+ charge states). Extended calibrants were used for mAb samples to cover any mAb oligomers.


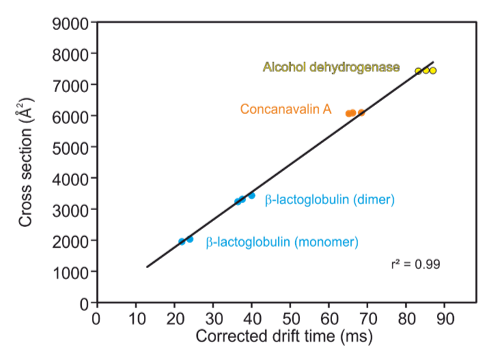


Figure S2: For Fab, Fc and I27 sample analysis, the TWIMS cell was calibrated using previously reported calibrant proteins (3). The plot shows: β-lactoglobulin monomer (7+ and 8+ charge states), β-lactoglobulin dimer (11+ to 13+ charge states), concanavalin A (19+ to 21+ charge states) and alcohol dehydrogenase (23+ to 25+ charge states).


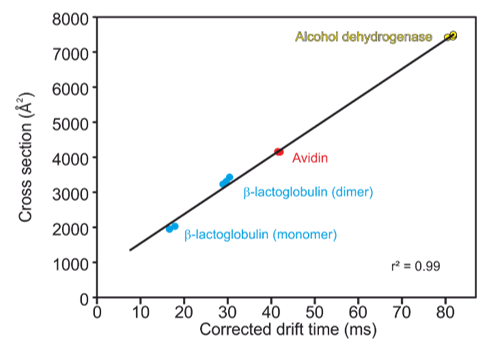


Figure S3: For POTRA and SasG sample analysis, the TWIMS cell was calibrated using previously reported calibrant proteins (3). The plot shows: β-lactoglobulin monomer (7+ and 8+ charge states), β-lactoglobulin dimer (11+ to 13+ charge states), avidin (15 to 17+ charge states) and alcohol dehydrogenase (23+ to 25+ charge states).

**ESI-TWIMS-MS Collision Cross-Section (CCS) calibrations for RNAs (4)**


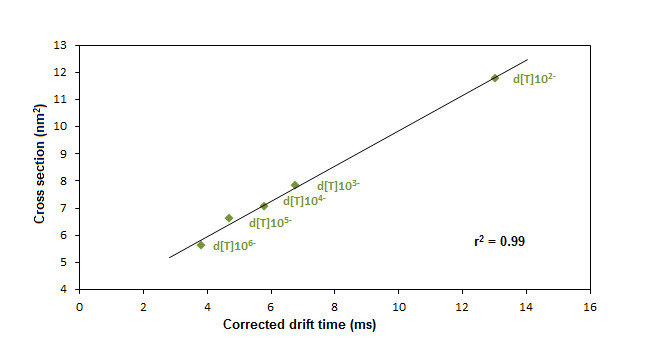


Figure S4: For RNA sample analysis, the TWIMS cell was calibrated using a previously reported calibrant oligonucleotide, d[T]_10_ (4). The plot shows: d[T]_10_ (2- to 6- charge states).

**References**

1. Emsley, P., Lohkamp, B., Scott, W. G., Cowtan, K.: Features and development of COOT. Acta Crystallogr. Section D: Biol. Crystallogr. 66, 486-501 (2010).
2. Ruotolo, B. T., Benesch, J. L., Sandercock, A. M., Hyung, S. J. Robinson, C. V.: Ion mobility-mass spectrometry analysis of large protein complexes. Nat. Protocols, **3**, 1139-1152 (2008).
3. Bush, M. F., Hall, Z., Giles, K., Hoyes, J., Robinson, C. V., Ruotolo, B. T.: Collision cross sections of proteins and their complexes: a calibration framework and database for gas-phase structural biology. Anal. Chem., **82**, 9557-9565 (2010).
4. Hoaglund, C.S., Liu, Y., Ellington, A.D., Pagel, M., Clemmer, D.E.: Gas-phase DNA: Oligothymidine ion conformers. J. Am. Chem. Soc., **119**, 9051–9052 (1997).
